# Computational discovery of high-temperature superconducting ternary hydrides via deep learning

**DOI:** 10.1093/nsr/nwag030

**Published:** 2026-01-16

**Authors:** Xiaoyang Wang, Chengqian Zhang, Zhenyu Wang, Hanyu Liu, Jian Lv, Han Wang, E Weinan, Yanming Ma

**Affiliations:** National Key Laboratory of Computational Physics, Institute of Applied Physics and Computational Mathematics, Beijing 100094, China; Academy for Advanced Interdisciplinary Studies, Peking University, Beijing 100871, China; Key Laboratory of Material Simulation Methods & Software of Ministry of Education and State Key Laboratory of Superhard Materials, College of Physics, Jilin University, Changchun 130012, China; International Center of Future Science, Jilin University, Changchun 130012, China; Key Laboratory of Material Simulation Methods & Software of Ministry of Education and State Key Laboratory of Superhard Materials, College of Physics, Jilin University, Changchun 130012, China; International Center of Future Science, Jilin University, Changchun 130012, China; Key Laboratory of Material Simulation Methods & Software of Ministry of Education and State Key Laboratory of Superhard Materials, College of Physics, Jilin University, Changchun 130012, China; National Key Laboratory of Computational Physics, Institute of Applied Physics and Computational Mathematics, Beijing 100094, China; Key Laboratory of High Energy Density Physics Simulation, Center for Applied Physics and Technology, School of Physics and College of Engineering, Peking University, Beijing 100871, China; AI for Science Institute, Beijing 100080, China; Center for Machine Learning Research, Peking University, Beijing 100871, China; School of Mathematical Sciences, Peking University, Beijing, 100871, China; Center for High-Pressure Science and Technology, Zhejiang University, Hangzhou 310027, China; School of Physics and Institute of Fundamental and Transdisciplinary Research, Zhejiang University, Hangzhou 310027, China

**Keywords:** large atom model, superconductor, machine learning, crystal-structure search

## Abstract

The discovery of novel high-temperature, or even room-temperature, superconducting materials holds transformative potential for a wide array of technological applications. However, the combinatorially vast chemical and configurational search space poses a significant challenge for both experimental and computational investigations. In this study, we employ the design of high-temperature ternary superhydride superconductors as a representative case to demonstrate how this challenge can be effectively addressed through a deep-learning-driven theoretical framework. This framework integrates high-throughput crystal-structure exploration, physics-informed screening and accurate prediction of superconducting critical temperatures. Our approach enabled the exploration of approximately 36 million ternary hydride structures across a chemical space of 29 elements, leading to the identification of 144 potential high-$T_{\rm c}$ superconductors with predicted $T_{\rm c} \ge 200$ K and superior thermodynamic stability at 200 GPa. Among these, 129 compounds spanning 27 novel structural prototypes are reported for the first time, representing a significant expansion of the known structural landscape for hydride superconductors. This work not only greatly expands the known repertoire of high-$T_{\rm c}$ hydride superconductors but also establishes a scalable and efficient methodology for navigating the complex landscape of multinary systems.

## Teaser

High-throughput crystal structure searches (CSP) performed by DPA-based large atom model (LAM) and Tc prediction model enabled the discovery of over 129 new superhydrides and 27 new prototypes.

## INTRODUCTION

The pursuit of superconductivity at elevated temperatures remains a fundamental and enduring challenge in condensed matter physics. While extensive research has explored a broad spectrum of superconducting materials, including unconventional systems with complex electronic correlations [1–3], the recent emergence of compressed binary hydrides as a prominent class of conventional superconductors with exceptionally high critical temperatures (*T*_c_) above 200 K has garnered considerable attention [4,5]. This surge of interest has been driven primarily by the experimental realization of superconductivity in $\mathrm{H}_{3}\mathrm{S}$ [6], as well as in a series of clathrate binary hydrides such as $\mathrm{CaH}_{6}$ [7,8], $\mathrm{YH}_{6}$ [9,10], $\mathrm{YH}_{9}$ [10] and $\mathrm{LaH}_{10}$ [11–13], which exhibit *T*_c_ values ranging from 215 to 260 K under high-pressure conditions. Notably, these discoveries were inspired by first-principles-based structure searching [14–19], underscoring the predictive capability of such theoretical approaches in materials design [20,21].

The exploration of superconductors is expanding from binary to ternary hydrides [4,5]. Predicted or synthesized non-stoichiometric ternary hydrides, such as (La,Y)H$_{10}$ ($T_{\rm c} \approx 253$ K at 183 GPa) [22], (La,Ce)H$_9$ ($T_{\rm c} \approx 148$–178 K at 97–172 GPa)[23] and (La,Al)H$_{10}$ ($T_{\rm c} \approx 223$ K at 164 GPa) [24], replicate the structural frameworks of binary hydrides such as CaH$_6$, YH$_9$ and LaH$_{10}$. In addition, stoichiometric ternary hydrides have been proposed through high-throughput structural substitution or crystal-structure prediction (CSP) [25–40]. Notable examples include LaBeH$_8$ [37], the first experimentally synthesized stoichiometric ternary hydride superconductor ($T_{\rm c} = 110$ K at 80 GPa) [41]; LaSc$_2$H$_{24}$, the first room-temperature superconductor ($T_{\rm c} = 298$ K at 260 GPa) [42]; recently synthesized $\mathrm{Y}_{0.5} \mathrm{Ce}_{0.5} \mathrm{H}_9$ ($T_{\rm c} = 129$ K at 114 GPa) [43]; $\mathrm{La}_{0.5}\mathrm{Ce}_{0.5}\mathrm{H}_{10}$ [44] ($T_{\rm c} = 175$ K at 155 GPa); the single-metal ternary $\mathrm{LaB}_{2}\mathrm{H}_{8}$ ($T_{\rm c} = 106$ K at 90 GPa) [45]; and theoretical predictions of compounds such as Li$_2$MgH$_{16}$[29] and NaLi$_3$H$_{23}$[30], which are expected to achieve exceedingly high *T*_c_ values under high pressure.

The immense number of possible stoichiometries and structural variations in ternary hydrides offers a promising avenue for enhancing *T*_c_. However, it also poses significant challenges for theoretical methods seeking to comprehensively explore and identify stable and superconducting phases. Traditional methods, such as high-throughput structural substitution [25,28,46], efficiently screen the compositional spaces but are limited to existing structural prototypes, which raises concerns about overlooking competing phases on the convex hull.

**Figure 1.**
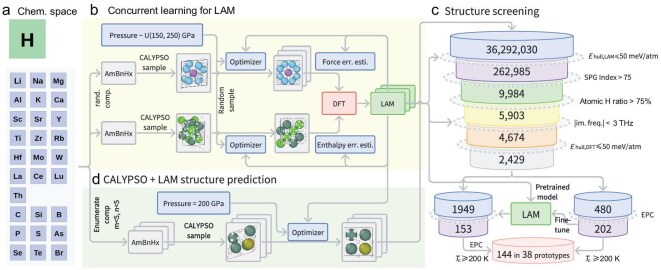
Schematic representation of the computational framework and workflows used in this study. (a) The chemical space covered in this work, comprising 29 elements: hydrogen, 19 metallic elements and 9 non-metallic elements. (b) Construction of the DPA based on a concurrent-learning scheme [47]. (c) Structure prediction using the CALYPSO method, with the DPA model employed for energy estimation during the large-scale exploration of ternary hydride structures. (d) Workflow for property screening, outlining the stepwise filtering criteria applied to identify high-temperature superconducting candidates.

First-principles-based CSP methods provide a comprehensive and template-free exploration of the configurational space [20,21]. However, their high computational cost limits their application to only a few ternary or multinary systems [29,30,36,37,39,40]. In addition, accurate *T*_c_ predictions require resource-intensive electron-phonon coupling calculations, further complicating the study of superhydrides. To address these challenges, we introduce here a deep-learning-driven theoretical framework for high-throughput discovery of high-temperature hydrogen-rich superconductors (Fig. 1).

Central to this framework is the large atomic model (LAM), a deep-learning model that consistently demonstrates high accuracy in characterizing the enthalpy landscape of ternary hydrides across a chemical space of 29 elements under high pressure. The framework incorporates the CALYPSO structure-prediction method [48,49], using the LAM as an enthalpy predictor to identify thermodynamically stable structures in a high-throughput manner. Subsequently, promising high-*T*_c_ candidates are screened using physics-informed filtering criteria and a *T*_c_-prediction model fine-tuned from the LAM. Using this framework, we explored approximately 36 million ternary hydride structures, leading to the identification of 144 potential high-temperature hydride superconductors across 38 structural prototypes. Notably, 129 of these hydrides, spanning 27 structural prototypes, represent novel predictions that, to the best of the authors’ knowledge, have not been reported previously in the literature.

## COMPUTATIONAL METHODS

### The LAM for compressed hydrides

The LAM for compressed hydrides explores a chemical space of 29 elements—hydrogen, 19 metallic elements and 9 non-metallic elements (Fig. 1a)—as previous studies suggest that their combinations with hydrogen may yield potential superconductors [4,5]. Based on the deep potential attention (DPA) architecture [50], the LAM was developed through two concurrent-learning iteration stages [47,51] (Fig. 1b). In the first stage, hydride structures ($\mathrm{A}_{m}\mathrm{B}_{n}\mathrm{H}_{x}$) were generated using the CALYPSO method and geometrically optimized under 150–250 GPa with the L-BFGS algorithm. Trajectory configurations exhibiting significant force errors were selected for labeling using density functional theory (DFT) and added to the training dataset iteratively until force predictions reached the desired accuracy. The subsequent stage enhanced the enthalpy-prediction accuracy for relaxed structures. The final dataset contained 218 349 samples with DFT calculated energy, force and virial values. The trained model achieved test accuracies of 37.5 meV/atom for energy, 171 meV/Å for forces and 45.7 meV/atom for virial predictions. Further details are provided in Section SI of the online supplementary material.

### DFT labeling

For dataset construction, the DFT calculations utilized a plane-wave basis set implemented in the ABACUS code [52,53]. The Perdew–Burke–Ernzerhof (PBE) exchange-correlation functional within the generalized gradient approximation [54] was used. Gaussian smearing with a width of 0.2 eV was applied. To ensure convergence of energy, force and virial-tensor calculations to within 0.001 eV/atom, 0.020 eV/Å and 0.020 eV/atom, respectively, an energy cutoff of 1360 eV and a Monkhorst–Pack *k*-point sampling grid spacing of 0.15 Å$^{-1}$ were selected. Optimized norm-conserving Vanderbilt pseudopotentials [55] generated by Schliph *et. al.* [56] were used for elements up to La (Z = 57) in our DFT calculations. For Ce, Lu and Th, pseudopotentials from PseudoDojo [57,58] were employed.

It is important to note that the DFT parameters employed for evaluating thermodynamic stability during structure screening differ slightly from those used in dataset construction. During the structure-screening procedure, DFT-based structure optimizations were performed using the conjugate-gradient method at 200 GPa. The Methfessel–Paxton smearing method with a smearing width of 0.2 eV was used. A Monkhorst–Pack *k*-point sampling grid spacing of 0.31 Å$^{-1}$ was applied.

### Electron-phonon coupling calculation

Electron-phonon coupling (EPC) calculations for superhydrides were performed using density functional perturbation theory [59] with the QUANTUM ESPRESSO package [60]. For these calculations, we employed SSSP PBE Precision v1.3.0 pseudopotentials [61], with a kinetic-energy cutoff of 816 eV for wave functions and 10 885 eV for the charge density and potential. For all the phonon calculations, *k*-meshes with a spacing of 0.24 Å^−1^ for electronic Brillouin-zone integration and *q*-meshes with a spacing of 0.48 Å^−1^ were used. Gaussian smearing with a width of 0.68 eV was applied. The superconducting gap and *T*_c_ were obtained by numerically solving the isotropic Eliashberg equation [62–64] using the ELK code [65].

### *T*_c_-prediction model

**Figure 2.**
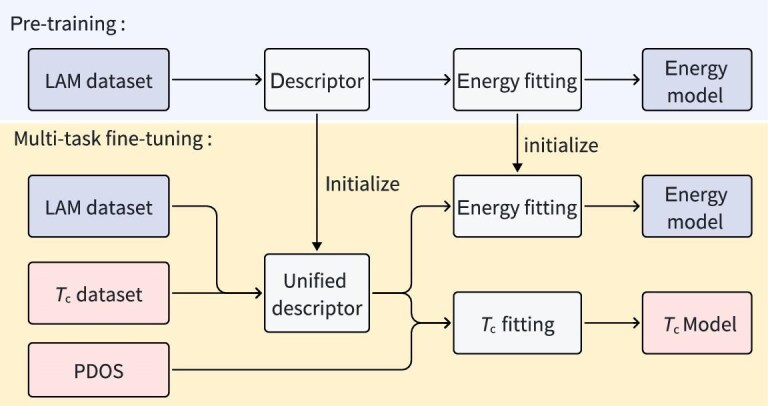
Schematic representation of the multi-task fine-tuning process for the *T*_c_-prediction model.

The construction of the *T*_c_-prediction model involved two integrated steps: pretraining the potential-energy LAM and multi-task fine-tuning of the *T*_c_-prediction model, as shown in Fig. 2. Both models were based on the DPA-2 architecture [66], with training conducted using the DeePMD-kit package [67]. The descriptor component of the pretrained model was used to initialize the corresponding component in the multi-task fine-tuning step. During this fine-tuning phase, the LAM was trained simultaneously on both the energy-prediction and *T*_c_-prediction tasks. Importantly, the DPA-2 descriptor maintained parameter sharing between these tasks, enabling synergistic learning by allowing structural knowledge from the potential-energy LAM to inform the *T*_c_-prediction model. In addition, the *T*_c_-prediction model incorporated hydrogen’s projected density of states (PDOS) at the Fermi level as a physically informed input to the fitting network, leveraging the established correlation between high hydrogen PDOS and elevated *T*_c_ in superhydrides [68–71].

The *T*_c_-prediction model exhibited a test mean absolute error (MAE) of $20.2 \pm 2.2$ K, as evaluated across four models. Each model was trained using distinct splits for the training and validation datasets, while consistently maintaining a 9:1 ratio between the training and validation datasets. The model achieved an MAE of 18.4 K in its predictions (see Section SII of the online supplementary material).

The *T*_c_-prediction model was subsequently employed to develop a dual-metric ranking algorithm, designed to prioritize our EPC calculations for candidate structures. Each configuration was ranked in ascending order according to its enthalpy above the convex hull, calculated using DFT and denoted as $R_{E_\mathrm{{hull,DFT}}}$. Simultaneously, configurations were ranked in descending order according to the model predicted *T*_c_, denoted as $R_{T_{\rm c}}$. The dual-metric rank is defined as


(1) \begin{eqnarray*}
R= \alpha \times R_{T_{\rm c}} + \beta \times R_{E_\mathrm{{hull,DFT}}},
\end{eqnarray*}

where *R* represents the overall ranking score, and $\alpha$ and $\beta$ are the relative weights assigned to each rank, both set to 0.5. The ranking score effectively balances the potential for a high *T*_c_ with thermodynamic stability. Candidates are sorted according to their ranking score *R*, and configurations with higher *R* values are selected for EPC validation of the transition temperature *T*_c_.

## RESULTS

### The high-throughput crystal-structure prediction

To achieve a comprehensive exploration of the material space for ternary hydrides, we systematically enumerated potential chemical compositions of ${\mathrm{A}_{m}\mathrm{B}_{n}\mathrm{H}_{x}}$ within the defined chemical parameters, where $m \le 4$, $n \le 4$ and $3(m+n) \le x \le 10(m+n)$. For each composition, CALYPSO structure predictions were performed at 200 GPa, with the LAM serving as the enthalpy-prediction workhorse. This approach enabled the exploration of 36 292 030 ternary hydride structures, ensuring comprehensive coverage of the targeted chemical space (Fig. 1c).

From this extensive dataset, a high-throughput screening workflow was developed to identify promising high-*T*_c_ superconductors with desirable stability (Fig. 1d).

1. *Thermodynamic stability (LAM level)*. Thermodynamic stability was evaluated using the LAM-predicted enthalpy above the convex hull ($E_{\mathrm{{hull,LAM}}}$). Hydrides with $E_{\mathrm{{hull,LAM}}} \le 50$ meV/atom were retained.
2. *Symmetry constraints*. Only high-symmetry hydrides—those with a space-group index above 75—were considered. This criterion prioritized orthogonal, trigonal, hexagonal and cubic systems.
3. *Atomic hydrogen fraction*. Atomic hydrogen, a key factor in hydride superconductivity, was quantified by identifying hydrogen atoms with interatomic distances exceeding 0.9 Å. Hydrides with an atomic-hydrogen fraction greater than 75% were retained.
4. *Dynamical stability*. Dynamical stability was ensured by filtering out hydrides with LAM-predicted imaginary phonon frequencies with magnitudes exceeding 3 THz.
5. *Thermodynamic stability (DFT level)*. Finally, the stability was re-evaluated using DFT, retaining hydrides with $E_{\mathrm{{hull,DFT}}} \le 50$ meV/atom.

The high-throughput structure search, coupled with a physics-driven screening workflow, generated a repository of 2429 candidate hydrides. This dataset provides a robust foundation for detailed EPC analyses, enabling the identification of promising high-*T*_c_ superconducting materials. However, performing EPC calculations for all the candidates remains computationally prohibitive. To overcome this challenge, a two-stage EPC analysis was implemented. In the first stage, EPC calculations (see the COMPUTATIONAL METHODS section) were performed for 480 of the 2429 hydrides. Priority was given to systems exhibiting high symmetry, small unit-cell sizes and a high hydrogen-dominated density of states at the Fermi level. This process resulted in 202 successful EPC calculations, yielding meaningful *T*_c_ results after excluding structures with computational errors or imaginary phonon frequencies. Notably, among these 202 hydrides, 100 candidates exhibited a calculated $T_{\rm c} > 200~\mathrm{K}$, distributed across 29 structural prototypes.

In the second stage, a *T*_c_-prediction model was fine-tuned from the energy-pretrained LAM, using data from the EPC calculations in the first stage (see the COMPUTATIONAL METHODS section). The model demonstrated a four-fold test mean absolute error of 20.2$\pm$2.2 K, 9.5 K ($\sim$30%) lower than a model trained from scratch. This improvement is attributed to the pretraining and multi-task fine-tuning strategy [66], which simultaneously fine-tunes the model on both the *T*_c_ and energy datasets, effectively mitigating catastrophic forgetting [72]. The remaining 1949 hydrides were ranked based on their $E_{\mathrm{{hull,DFT}}}$ and predicted *T*_c_. The top-ranked candidates were subsequently subjected to detailed EPC calculations to further assess their potential as high-*T*_c_ superconductors. At this stage, 153 hydrides were analyzed, with 57 *T*_c_ values successfully obtained, with 44 compounds across 14 structural prototypes identified as exhibiting *T*_c_ exceeding 200 K.

### The discovered high-*T*_c_ hydrides

**Figure 3.**
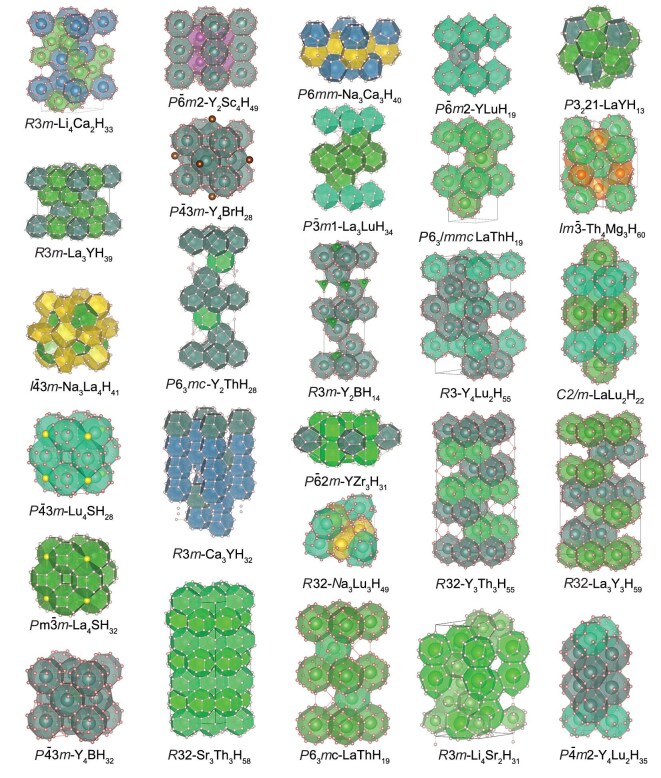
Representative crystal structures of the 27 newly predicted ternary hydride prototypes.

The proposed theoretical framework has collectively identified 144 potential high-temperature hydride superconductors across 38 distinct structural prototypes at 200 GPa, all with predicted *T*_c_ exceeding 200 K. Importantly, all of them exhibit good thermodynamic stability ($E_{\mathrm{{hull,DFT}}} \le 50$ meV/atom). Of these, 129 hydrides and 27 structural prototypes represent novel predictions that, to the best of our knowledge, have not been previously reported in the literature. While previous studies reported $\sim 30$ clathrate hydride prototypes [46,73], the 27 new prototypes, introduced by this work nearly double the number of known clathrate prototypes for high-*T*_c_ hydride superconductors. Representative crystal structures of the superhydrides adopting these 27 new prototypes are illustrated in Fig. 3.

**Figure 4.**
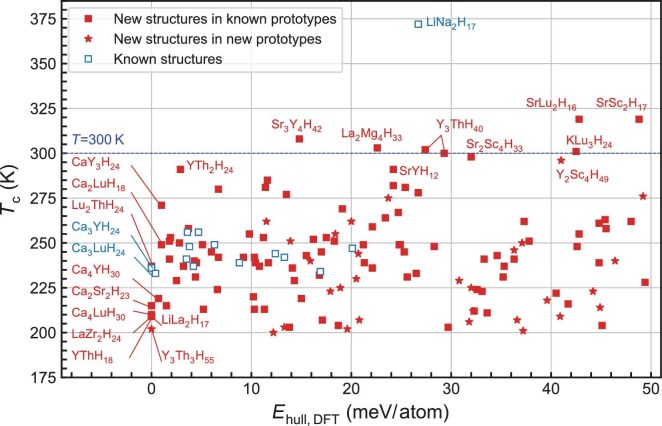
Scatter plot of the 144 predicted ternary hydride superconductors in *T*_c_-$E_\mathrm{{hull,DFT}}$ space at 200 GPa. Previously reported compounds reproduced in this study are represented by open blue squares, while newly predicted compounds are shown as filled red squares. Compounds adopting novel structural prototypes are highlighted with red stars.

The scatter plot of the 144 predicted hydride superconductors in *T*_c_-$E_{\mathrm{{hull,DFT}}}$ space at 200 GPa is shown in Fig. 4. A complete list, categorized by structural prototypes, is provided in Section SIII of the online supplementary material. Twelve hydrides are predicted to exhibit *T*_c_ approaching or exceeding room temperature. These compounds include $R\bar{3}m$-${\mathrm{Sr}_{3}\mathrm{Y}_{4}\mathrm{H}_{42}}$ ($T_{\rm c} \approx 308$ K), $Fm \bar{3}m$-${\mathrm{KLu}_{3}\mathrm{H}_{24}}$ ($T_{\rm c} \approx 301$ K), $Fd \bar{3}m$-${\mathrm{SrYH}_{12}}$ ($T_{\rm c} \approx 291$ K), $Pm \bar{3}m$-${\mathrm{Y}_{3}\mathrm{ThH}_{40}}$ ($T_{\rm c} \approx 302$ K), $R \bar{3}m$-${\mathrm{Y}_{3}\mathrm{ThH}_{40}}$ ($T_{\rm c} \approx 300$ K), $P6/mmm$-$\mathrm{Y}_{2}\mathrm{ThH}_{24}$ ($T_{\rm c} \approx 291$ K), $Fd \bar{3}m$-$\mathrm{SrLu}_{2}\mathrm{H}_{16}$ ($T_{\rm c} \approx 319$ K), $Fd \bar{3}m$-$\mathrm{Li}_{2}\mathrm{NaH}_{17}$ ($T_{\rm c} \approx 372$ K), $Fd \bar{3}m$-$\mathrm{SrSc}_{2}\mathrm{H}_{17}$ ($T_{\rm c} \approx 319$ K), $F \bar{4}3m$-$\mathrm{La}_{2}\mathrm{Mg}_{4}\mathrm{H}_{33}$ ($T_{\rm c} \approx 303$ K), $F\bar{4}3m$-$\mathrm{Sr}_{2}\mathrm{Sc}_{4}\mathrm{H}_{33}$ ($T_{\rm c} \approx 298$ K) and $P\bar{6}m2$-$\mathrm{Y}_{2}\mathrm{Sc}_{4}\mathrm{H}_{49}$ ($T_{\rm c} \approx 296$ K). Notably, only $Fd\bar{3}m$-$\mathrm{Li}_{2}\mathrm{NaH}_{17}$ and $P6/mmm$-$\mathrm{Y}_{2}\mathrm{ThH}_{24}$ have been previously reported, underscoring the predictive power and efficacy of the theoretical framework in discovering novel high-*T*_c_ hydride superconductors. Detailed results of the EPC calculation of these compounds are presented in Section SIV of the online supplementary material.

Eight predicted hydrides are identified as lying on the convex hull, signifying their thermodynamic stability and high potential for experimental synthesis. These include $Fm\bar{3}m$-${\mathrm{Ca}_{3}\mathrm{YH}_{24}}$ ($T_{\rm c} \approx 236$ K), $R \bar{3}m$-$\mathrm{Ca}_{4}\mathrm{LuH}_{30}$ ($T_{\rm c} \approx 210$K), $P6/mmm$-$\mathrm{Lu}_{2}\mathrm{ThH}_{24}$ ($T_{\rm c} \approx 237$K), $P6/mmm$-$\mathrm{LaZr}_{2}\mathrm{H}_{24}$ ($T_{\rm c} \approx 209$K), $P\bar{6}m2$-$\mathrm{YThH}_{18}$ ($T_{\rm c} \approx 209$K), $Fd \bar{3}m$-$\mathrm{Ca}_{2}\mathrm{Sr}_{2}\mathrm{H}_{23}$ ($T_{\rm c} \approx 215$ K), $I4/mmm$-$\mathrm{LiLa}_{2}\mathrm{H}_{17}$ ($T_{\rm c} \approx 209$ K) and $R32$-$\mathrm{Y}_{3}\mathrm{Th}_{3}\mathrm{H}_{55}$ ($T_{\rm c} \approx 202$K). Among them, only $Fm \bar{3}m$-${\mathrm{Ca}_{3}\mathrm{YH}_{24}}$ have been previously proposed. It is noteworthy that, while the remaining hydrides are predicted to be metastable, they exhibit only marginal deviations from thermodynamic stability ($E_\mathrm{{hull,DFT}}\le 50$ meV/atom), which suggests a likelihood of their synthesis [74]. Furthermore, these compounds could potentially be stabilized at pressures different from those considered here. For example, several hydrides in the Ca-Y-H, Ca-Lu-H and Y-Lu-H systems, calculated to be marginally metastable ($E_\mathrm{{hull,DFT}}\le 10$ meV/atom at 200 GPa; Section III of the online supplementary material), could be stabilized at higher pressures [26–28]. The $Fd \bar{3}m$-$\mathrm{Li}_{2}\mathrm{NaH}_{17}$, which exhibits an exceptionally high *T*_c_ of 372 K and $E_\mathrm{hull}= 26.7$ meV/atom at 200 GPa, is predicted to become thermodynamically stable at higher pressures [30]. Moreover, other effects, such as anharmonicity, were not considered due to computational cost, though they may influence stability pressure or superconductivity [40]. Further investigations into the impact of these effects on the thermodynamic stability of the predicted compounds at different pressures are warranted for future studies.

Several chemical systems have been identified as hosting a substantial number of compounds that satisfy the screening criteria $T_{\rm c} \ge 200$ K and $E_\mathrm{{hull,DFT}}\le 50$ meV/atom, as shown in Table 1. These systems emerge as strong candidates for prioritization in experimental validations due to their considerable potential for the discovery of novel high-*T*_c_ superconductors. In particular, the Y-Lu-H, Ca-Y-H, Ca-Lu-H, Sr-Y-H, Na-Y-H and Y-La-H systems predominantly feature hydrides with $\mathrm{CaH}_{6}$-type structures. These structures are distinguished by variations in the occupation of A and B atoms within body-centered cubic (bcc) metal lattices. Moreover, this class of structures generally exhibits lower $E_\mathrm{{hull,DFT}}$ values compared to other structural prototypes, suggesting enhanced thermodynamic stability. Consequently, these systems are promising candidates for the experimental synthesis of non-stoichiometric alloy hydrides, as supported by prior experimental findings [22].

**Table 1.** The number of structures with $T_{\rm c} \ge 200$K in each ternary system and their distribution across structural prototypes (#proto.). For each ternary system, configurations with $T_{\rm c} \ge 250$K are explicitly listed for comparison.

System	#($T_{\rm c}\ge 200$K)	#proto.	#($T_{\rm c} \ge 250$K)
Y-Lu-H	15	4	1
Ca-Y-H	13	2	7
Ca-Lu-H	12	1	1
Y-Th-H	10	6	4
Sr-Y-H	7	2	7
Na-Y-H	6	1	1
Y-La-H	5	3	3
Ca-Sc-H	4	1	1
La-Th-H	4	3	0
Na-Lu-H	4	2	0
Sc-Sr-H	4	4	3

**Figure 5.**
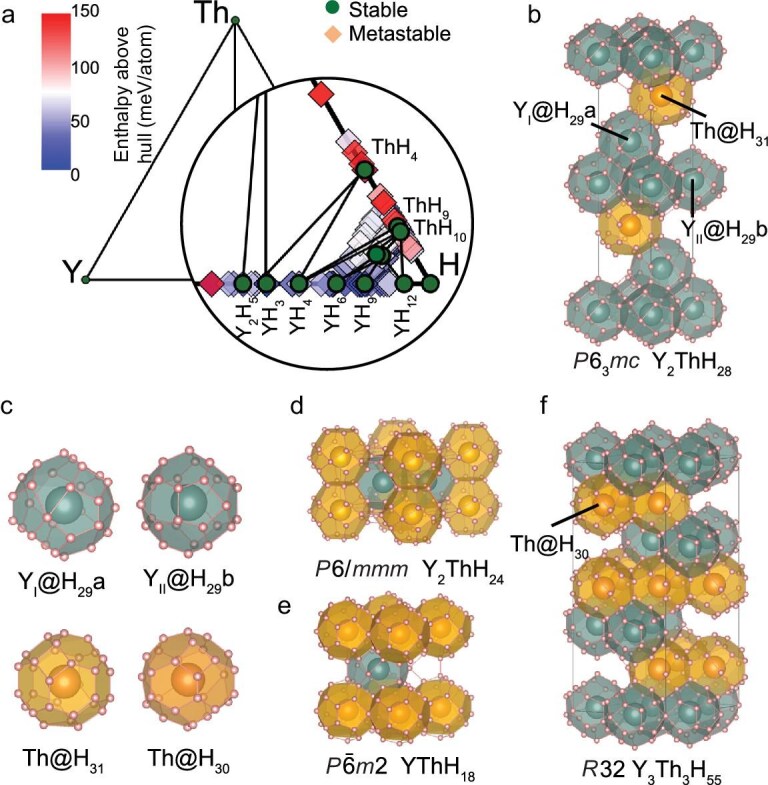
The thermodynamic stability and crystal structures of representative hydrides in the Y-Th-H ternary system. (a) The ternary convex hull for the Y-Th-H system. (b) The crystal structure of $P6_3/mc$-$\mathrm{Y}_{2}\mathrm{ThH}_{28}$. (c) The detailed configurations of the two Y-centered $\mathrm{H}_{29}$ cages, $\mathrm{H}_{29}$a and $\mathrm{H}_{29}$b, a Th-centered $\mathrm{H}_{31}$ cage found in $\mathrm{Y}_{2}\mathrm{ThH}_{28}$ and a Th centered $\mathrm{H}_{30}$ cage found in $\mathrm{Y}_{3}\mathrm{Th}_{3}\mathrm{H}_{55}$. The $\mathrm{H}_{29}$a and $\mathrm{H}_{31}$ cages are novel findings in $\mathrm{Y}_{2}\mathrm{ThH}_{28}$, while $\mathrm{H}_{29}$b is the typical $\mathrm{H}_{29}$ cage found in $\mathrm{YH}_{9}$-type structures. (d) The crystal structure of $P6/mmm$-$\mathrm{Y}_{2}\mathrm{ThH}_{24}$, (e) $P\bar{6}m2$-$\mathrm{YThH}_{18}$ and (f) $R32$-$\mathrm{Y}_{3}\mathrm{Th}_{3}\mathrm{H}_{55}$.

The Y-Th-H system emerges as a particularly notable discovery, encompassing 10 hydrides spanning six structural prototypes that meet the screening criteria. Interestingly, structural prototypes in this system, apart from the conventional $\mathrm{CaH}_{6}$ and $\mathrm{LaH}_{10}$, exhibit superior thermodynamic stability. The enthalpy convex hull for the Y-Th-H system at 200 GPa is presented in Fig. 5a. The $P6_3mc$-$\mathrm{Y}_{2}\mathrm{ThH}_{28}$, as shown in Fig. 5b, adopts a novel structural prototype featuring two types of Y-centered $\mathrm{H}_{29}$ cages and a Th-centered $\mathrm{H}_{31}$ cage, as detailed in Fig. 5c. This compound demonstrates $T_{\rm c} \approx 255$ K and $E_\mathrm{{hull,DFT}}= 18.4$ meV/atom. The $P6/mmm$-$\mathrm{Y}_{2}\mathrm{ThH}_{24}$, as shown in Fig. 5d, shares the same structural prototype as the recently proposed $P6/mmm$-$\mathrm{LaSc}_{2}\mathrm{H}_{24}$ [40] and exhibits a calculated $T_{\rm c} \approx 291$ K, approaching room temperature. This compound lies only 2.9 meV/atom above the hull and achieves thermodynamic stability at 170 GPa [46]. In addition, two thermodynamically stable compounds, $P\bar{6}m2$-$\mathrm{YThH}_{18}$ and $R32$-$\mathrm{Y}_{3}\mathrm{Th}_{3}\mathrm{H}_{55}$, have been identified, with calculated *T*_c_ of 209 and 202 K, respectively. The $P\bar{6}m2$-$\mathrm{YThH}_{18}$, depicted in Fig. 5e, adopts the $\mathrm{YH}_{9}$-type structure, in which Y and Th atoms are arranged in a hexagonal close-packed (hcp) lattice. The $R32$-$\mathrm{Y}_{3}\mathrm{Th}_{3}\mathrm{H}_{55}$, shown in Fig. 5f, represents a novel structural prototype composed of a network of Y-centered $\mathrm{H}_{29}$ cages, previously found in $\mathrm{YH}_{9}$[15], and a new Th-centered $\mathrm{H}_{30}$ cage, as shown in Fig. 5c. The presence of these structures demonstrates that the Y-Th-H system holds great promise for the discovery of stoichiometric ternary hydrides with novel structural types and high-temperature superconductivity.

## CONCLUSIONS

In summary, we propose a deep-learning-driven computational framework to address the challenges of exploring the vast chemical and configurational space in materials discovery. By combining high-throughput global structure searching, physics-guided screening and first-principles-based property calculations—enhanced by a deep-learning-based LAM—the framework serves as a powerful tool for accelerating the discovery of novel materials. Applied to ternary superhydrides, the framework has led to the discovery of 129 compounds with superconducting transition temperatures exceeding 200 K. This investigation unveiled a diverse range of structural prototypes, encompassing both previously known clathrate-like configurations and entirely new structures, almost doubling the number of known clathrate prototypes of high-*T*_c_ hydride superconductors. These results underscore the predictive power and efficiency of the proposed approach in navigating the intricate landscape of multinary hydrides.

Future work will focus on extending this framework to quaternary and higher-order hydride systems, while systematically examining the effects of pressure variations on their stability and superconducting properties. Experimental validation will be crucial to confirm the predicted structures and *T*_c_ values, as well as to provide deeper insights into the mechanisms underlying superconductivity. Overall, this study establishes a robust foundation for advancing high-temperature superconductivity, with far-reaching implications for both fundamental research and practical applications.

## Supplementary Material

nwag030_Supplemental_File
