# Supplementary material for: Computational discovery of high-temperature superconducting ternary hydrides via deep learning
Source: Natl Sci Rev. 2026 Jan 16;13(6):nwag030. doi: 10.1093/nsr/nwag030 (PMC13023052; doi:10.1093/nsr/nwag030)
Supplement: nwag030_Supplemental_File [file nwag030_supplemental_file.pdf]

Supporting Information of "Computational discovery of High-Temperature Superconducting Ternary Hydrides via Deep Learning"

Xiaoyang Wang,[1] Chengqian Zhang,[2] Zhenyu Wang,[3,4] Hanyu Liu,[3,4,*]
Jian Lv,[3,*] Han Wang,[1,5,*] Weinan E,[6,7,8] and Yanming Ma[3,4,*]
[1]*National Key Laboratory of Computational Physics, Institute of Applied Physics and Computational Mathematics, Fenghao East Road 2, Beijing 100094, P.R. China*
[2]*Academy for Advanced Interdisciplinary Studies, Peking University, Beijing 100871, P. R. China*
[3]*Key Laboratory of Material Simulation Methods & Software of Ministry of Education and State Key Laboratory of Superhard Materials, College of Physics, Jilin University, Changchun 130012, P.R. China*
[4]*International Center of Future Science, Jilin University, Changchun 130012, P.R. China*
[5]*HEDPS, CAPT, College of Engineering, Peking University, Beijing 100871, P.R. China*
[6]*AI for Science Institute, Beijing 100080, China*
[7]*Center for Machine Learning Research, Peking University, Beijing 100871, P.R. China*
[8]*School of Mathematical Sciences, Peking University, Beijing, 100871, P.R. China*
(Dated: November 11, 2025)

Section SI. LAM construction

The construction of training datasets for the 29-element (LAM) ternary superhydrides was accomplished using the concurrent learning method [1], as implemented by the Deep Potential Generator (DP-GEN) framework [2]. Beginning with an initial training dataset, the quality of the LAM model is iteratively refined. In each iteration, an ensemble of LAM models is trained using the current training dataset. Subsequently, one LAM model from the ensemble is utilized to explore the configuration and chemical space via the CLYPSO CSP algorithm. Configurations that exhibit significant errors, as estimated by the ensemble of LAM models, are labeled through DFT calculations. The labeled data are then integrated into the training dataset to initiate the next iteration. This methodology has been demonstrated to be effective in developing CSP-oriented machine-learning interatomic potential models [1].

Constraints and Initial Dataset

Ternary hydride structures, represented by the chemical formula $\mathrm{A_mB_nH_x}$, were generated using the CALYPSO software. In this formula, A and B denote non-hydrogen elements, while m and n ($1 \leq m, n \leq 4$) specify the number of A and B atoms within a unit cell. The number of hydrogen atoms, x, was systematically determined based on the chemical types and quantities of A and B. Specifically, when both A and B are metals, x was designated as an integer ranging from 5 to 10 times the sum of m and n. In instances where both A and B are non-metals, x was set as an integer 3 to 5 times the sum of m and n. When one element is a metal and the other a non-metal, x was determined as an integer between 3 and 10 times the sum of m and n. To ensure a balanced representation of chemical diversity, the generated configurations were distributed as follows: 50% of the cases involved both A and B being metals, while 25% corresponded to both being non-metals, and the remaining 25% involved one metal and one non-metal.

Under these constraints, we generated 20,000 initial configurations and labeled them using single-point DFT calculations. These structures, along with the DFT labels for energies, atomic forces, and virial tensors, constitute the initial dataset. This dataset serves as the foundation for constructing datasets in subsequent iterations.

To prevent CALYPSO from generating atomic structures with excessively short interatomic distances, which could lead to the exploration of non-physical structures, we introduced a "safe radius" (r_s) for each element, as detailed in Table **SI**. These safe radii [3] were used to calculate the minimum allowable interatomic distance between a pair of atoms A and B in the dataset:

$$d_{min} = \alpha(r_s(\mathrm{A}) + r_s(\mathrm{B}))/1.2, \tag{1}$$

* Corresponding Author: Hanyu Liu, hanyuliu@jlu.edu.cn; Jian Lv, lvjian@jlu.edu.cn; Han Wang, wang_han@iapcm.ac.cn; Yanming Ma, mym@jlu.edu.cn

Table SI. Safe radii (r_s) of elements used for calculating minimum allowable interatomic distances.

-	Element	r_s (Å)	Element	r_s (Å)
Metals	Li	0.794		
	Na	0.767	La	1.323
	K	1.217	Ti	1.058
	Rb	1.323	Zr	1.111
	Mg	0.899	Hf	1.270
	Ca	1.217	Mo	1.111
	Sr	1.323	W	1.217
	Al	0.899	Ce	1.349
	Sc	1.058	Lu	1.481
	Y	1.111	Th	1.481
Non-metals	B	0.582	S	0.794
	C	0.582	Se	1.111
	Si	0.846	Te	1.058
	P	0.794	Br	1.111
	As	1.058	**H**	**0.432**

where α is an adjustable prefactor. During the generation of initial configurations using CALYPSO, the prefactor α was set to 1.3 for pairs consisting of hydrogen and non-hydrogen atoms, and 1.5 for pairs of non-hydrogen atoms. The criteria did not apply to pairs of H atoms; instead, any pair with a distance smaller than 1.0 Å was excluded.

Training

The LAM utilized in this study was based on the DPA-1 architecture [4], with training conducted using the DeePMD-kit package [5]. In each iteration, four models were trained using identical training data and hyperparameters, with the parameters of each model initialized with a distinct random seed. The cutoff radius was set at 6 Å. The number of neurons in the embedding network was [25, 50, 100], and number of neurons in the fitting network was [240, 240, 240]. Training was performed using the Adam stochastic gradient descent method [6]. The loss function was defined by

$$\mathcal{L} = \frac{1}{|\mathcal{B}|}\sum_{k\in\mathcal{B}}\Big(p_\epsilon \frac{1}{N}|\hat{E}^k - E^k|^2 + p_f\frac{1}{3N}\sum_{i\alpha}|\hat{F}^k_{i\alpha} - F^k_{i\alpha}|^2 + p_\xi\frac{1}{9N}\sum_{i\alpha}|\hat{\Xi}^k_{\alpha\beta} - \Xi^k_{\alpha\beta}|^2\Big), \quad (2)$$

where $|\mathcal{B}|$ was the size of mini-batch. In the loss function, E^k, $F^k_{i\alpha}$, and $\Xi^k_{\alpha\beta}$ represent the model-predicted energy, force, and virial tensor for the k-th configuration, respectively, and $\hat{E}^k$, $\hat{F}^k_{i\alpha}$, and $\hat{\Xi}^k_{\alpha\beta}$ denote the energy, forces, and virial tensors calculated using DFT for the same configuration. N denotes the number of atom in a data frame. The prefactors (p_ϵ, p_f, p_ξ) are hyperparameters that dictate the relative significance of the energy, forces, and virial tensor during training. These prefactors are progressively adjusted in accordance with the learning rate, which undergoes exponential decay as the training progresses.

During the concurrent learning iterations, the models are trained for 400,000 steps. The learning rate begins at 2×10^{-4} and exponentially decreases to 3.51×10^{-8} over the course of the training. Initially, the prefactors (p_ϵ, p_f, p_ξ) are set to (0.02, 1000, 0.0) are linearly adjusted in relation to the learning rate to (1.0, 1.0, 0.0) by the end of the training. Once the size of the training dataset surpasses 100,000 entries, model parameters are initialized using the models from the previous iteration. In this case, the initial learning rate is reduced to 1×10^{-4}, and the initial prefactors (p_ϵ, p_f) are modified to (0.2, 100).

Iterations to Reduce the Force Error

In each of the first three iterations, 500,000 ternary superhydride configurations were generated using the CALYPSO algorithm. The criteria for minimal atomic distance were maintained consistent with those established for generating the initial dataset. The maximum standard deviation of the predicted atomic forces by the model ensemble, termed force deviation, was then determined. Configurations with a force deviation exceeding 1.0 eV/Å were initially excluded because they are potentially nonphysical. The remaining configurations were sorted by force deviation, and 10,000 configurations were randomly sampled from the top 10 % for DFT calculations. By the end of the third iteration, the minimum force deviation within this top 10th percentile was 410 meV/Å.

Table SII. The RMSE of LAM on the training and testing datasets.

Dataset	Size	$RMSE_E$ (meV/atom)	$RMSE_F$ (meV/Å)	$RMSE_V$ (meV/atom)
Training	218,349	48.1	334	83.9
Testing	1,649	37.5	171	45.7

In each of the subsequent three iterations, 100,000 configurations were generated using the CALYPSO algorithm and subsequently optimized using one model from the ensemble. The criteria for configuration generation were identical to those used for the initial dataset. The optimization was performed using the L-BFGS algorithm as implemented in the Atomic Simulation Environment (ASE) package [7], with the maximum number of steps restricted to 20. The convergence criterion for forces was set at 20 meV/Å, and the target pressure for optimization was randomly sampled between 150 GPa and 250 GPa. force deviations were calculated for the 1st, 5th, 10th, last 10th, last 5th, and final configuration along each optimization trajectory. In total, 10,000 configurations were sampled following the same criteria applied in the initial three iterations. Configurations that violated the criterion specified in Eq. (1) with $\alpha = 1$ were excluded from this selection. In the following thirteen iterations, the number of structural optimization steps was relaxed to 200.

In certain known superhydrides, such as SrH_{22} [8], MgH_{12}, and ScH_{12} [9],hydrogen atoms exist as H_2 molecules or quasi-molecules, with the nearest H-H distance being less than 1.0 Å. In previous iteration stages, configurations of this type were deliberately excluded to enhance data quality, although exploration of such structures remains necessary. Consequently, we adjusted the closest distance criterion for configuration generation to $\alpha = 1.0$ for all elements and conducted an additional four iterations with 200 steps of structural optimization. Finally, the force deviation at the top 10th percentile converged to 360 meV/Å.

Iterations to Improve the Enthalpy Accuracy

In each iteration, 500,000 configurations were generated by CALYPSO algorithm and subsequently optimized using one model from the ensemble. The standard deviations of the predicted per-atom enthalpies by the model ensemble were calculated for the final configuration of each optimization trajectory and then sorted. From the top 10% of these configurations, 40,000 configurations that met the criterion in Eq. (1) with $\alpha = 1$ were randomly sampled for DFT calculations. By the end of the 4th iteration, the enthalpy deviation within the top 10th percentile had converged to 22 meV/atom.

The productive LAM

The concurrent learning scheme produced a training dataset comprising 218,349 labeled structures. Utilizing this dataset, the productive LAM was ultimately trained from scratch over 16,000,000 steps, with the learning rate decaying from 2×10^{-4} to 3.51×10^{-8}. The initial prefactors (p_ϵ, p_f, p_ξ) in the loss function were set to (0.02, 1000, 0.02), and the final prefactors were adjusted to (1.0, 1.0, 1.0). The training and testing RMSEs for energy, force, and virial tensors of the productive LAM are reported in Table **SII**. The test dataset is an independent collection comprising 1,649 DFT-relaxed configurations.

Section SII. The accuracy of the T_c prediction model

The training, test, and prediction errors of the T_c prediction model are shown in Figure **S1**(a)-(c). Among the 153 configurations screened by the T_c model, 57 were confirmed to be dynamically stable, and their T_c values were successfully obtained through DFT calculations. The majority of these values exceed 200 K, and the T_c prediction model achieves a mean absolute error of 18.40 K on its predictions.

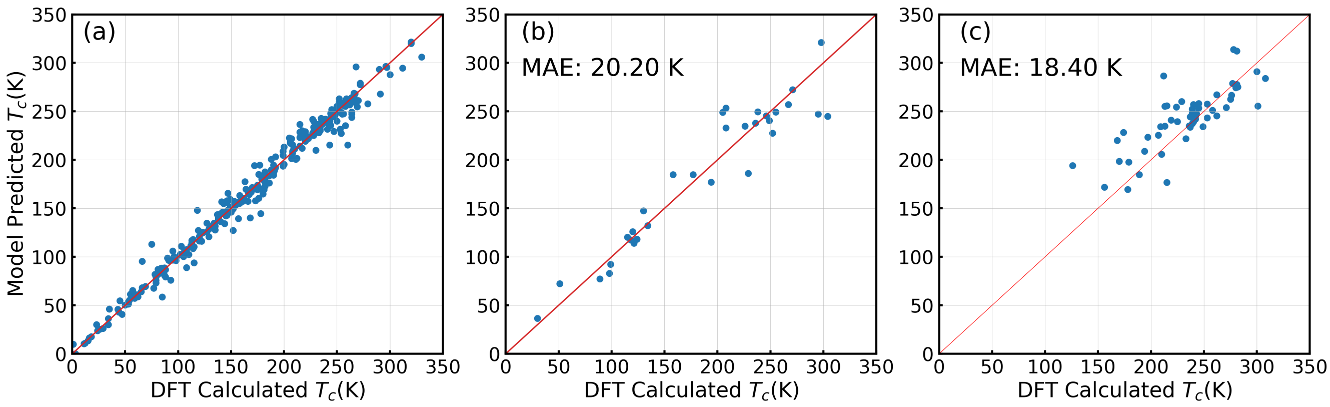

Figure S1. The comparison between the T_c prediction model results and DFT results on (a) the training dataset. (b) the test dataset (c) the prediction results.

Section SIII. Summary of predicted high-T_c superhydrides.

The complete list of predicted ternary hydrides with $T_c \geq 200$ K at 200 GPa, categorized by their structural prototypes, is provided in Supporting Table **SIII**. Hydrides within the same prototype are characterized by an identical hydrogen framework, with variations in the occupation of A and B atoms at lattice sites typically associated with metal atoms. Prototypes are labeled based on either the chemical formulas of representative compounds or generalized formulas ($A_mB_nH_x$). Prototypes that share the same generalized formulas are distinguished by their space groups. For example, the newly discovered $I4/mcm$ $Li_1La_2H_{17}$ adopts a different prototypes from the known structure $Fd\bar{3}m$ $Li_2La_1H_{17}$ [10].

Table SIII. The full list of 144 ternary hydride superconductors predicted in this study, organized by their structural prototypes. For each compound, the space group symmetry, enthalpy above the convex hull ($E_{\mathrm{hull,DFT}}$), and the calculated superconducting critical temperature (T_c) at 200 GPa are provided. Prototypes marked with * indicate those that have been previously reported. T_cs are calculated by numerically solving the isotropic Eliashberg equation, assuming $\mu^* = 0.10$. All structure files in POSCAR format are provided in `https://www.aissquare.com/datasets/detail?pageType=datasets&name=144HighTcSuperhydridesPOSCAR&id=315`.

Prototype	Composition	Space Group	$E_{\mathrm{hull,DFT}}$ (meV/atom)	T_c (K)	Prototype	Composition	Space Group	$E_{\mathrm{hull,DFT}}$ (meV/atom)	T_c (K)
CaH_6 type*	$Sr_3Y_4H_{42}$	$R\bar{3}m$	14.8	308	CaH_6 type*	$NaYH_{12}$	$Fd\bar{3}m$	1.5	215
	KLu_3H_{24}	$Fm\bar{3}m$	42.5	301		NaY_2H_{18}	$P\bar{3}m1$	5.2	213
	$SrYH_{12}$	$Fd\bar{3}m$	24.2	291		Ca_4ScH_{30}	$R\bar{3}m$	11.3	213
	SrY_3H_{24}	$Fm\bar{3}m$	11.6	285		Ca_3ThH_{24}	$Fm\bar{3}m$	33.6	211
	KY_3H_{24}	$P4/mmm$	24.2	282		Ca_4LuH_{30}	$R\bar{3}m$	0	210
	$Sr_2Y_3H_{30}$	$R\bar{3}m$	11.4	281		Ca_4LaH_{30}	$P\bar{1}$	17.1	207
	SrY_3H_{24}	$P4/mmm$	6.7	280		$NaLuH_{12}$	$Fd\bar{3}m$	13.8	203
	SrY_2H_{18}	$C2/m$	13.5	277	LaH_{10} type*	Y_3ThH_{40}	$Pm\bar{3}m$	27.4	302
	CaY_3H_{24}	$P4/mmm$	1.0	271		Y_3ThH_{40}	$R\bar{3}m$	29.3	300
	KY_4H_{30}	$R\bar{3}m$	19.1	269		SrY_3H_{40}	$R32$	26.7	278
	KY_3H_{24}	$Fm\bar{3}m$	24.7	267		$RbTh_3H_{40}$	$Pm\bar{3}m$	42.8	255
	$La_2Y_3H_{30}$	$R\bar{3}m$	23.4	264		La_3CeH_{40}	$Pm\bar{3}m$	11.2	253
	Y_3MgH_{24}	$Fm\bar{3}m$	45.4	263		KTh_3H_{40}	$Pm\bar{3}m$	34.6	243
	$SrLu_3H_{24}$	$Fm\bar{3}m$	37.3	262		$La_4Th_3H_{70}$	$R3$	26.5	233
	$CaScH_{12}$	$Fd\bar{3}m$	44.8	261		Y_3ThH_{40}	$R3$	33.1	223
	LaY_3H_{24}	$Fm\bar{3}m$	22.1	259		$SrTh_3H_{40}$	$Pm\bar{3}m$	10.3	213
	$CaYH_{12}$	$P4/nmm$	3.7	258		$CaTh_3H_{40}$	$Pm\bar{3}m$	29.7	203
	$Ca_1Y_3H_{24}$[11]	$Fm\bar{3}m$	3.6	256	AB_2H_{24}*	Y_2ThH_{24}[12]	$P6/mmm$	2.9	291
	$CaLuH_{12}$[13]	$Pm\bar{3}m$	4.7	256		YZr_2H_{24}	$P6/mmm$	25.3	245
	$Na_1Y_3H_{24}$	$Fm\bar{3}m$	9.8	255		Lu_2ThH_{24}	$P6/mmm$	0	237
	$Ca_3Y_4H_{42}$	$R\bar{3}m$	1.9	253		$ThSc_2H_{24}$	$P6/mmm$	2.5	229
	Ca_4LaH_{30}	$R\bar{3}m$	17.5	253		$LaZr_2H_{24}$	$P6/mmm$	0	209
	LaY_2H_{18}	$P\bar{3}m1$	18.3	251		$CeLu_2H_{24}$	$P6/mmm$	18.7	204
	CaY_4H_{30}	$R\bar{3}m$	1.8	251	AB_2H_{16}*	$SrLu_2H_{16}$	$Fd\bar{3}m$	42.8	319
	$Ca_3Y_2H_{30}$	$R\bar{3}m$	2.8	250		$LaSc_2H_{16}$	$Fd\bar{3}m$	48.0	262
	Y_3ZrH_{24}	$Fm\bar{3}m$	21.2	249		$CeSc_2H_{16}$	$Fd\bar{3}m$	28.3	248
	Y_3LuH_{24}[13]	$Fm\bar{3}m$	6.3	249		$SrSc_2H_{16}$	$Fd\bar{3}m$	10.2	220
	$NaLu_3H_{24}$	$Fm\bar{3}m$	24.8	249		$SrTi_2H_{16}$	$Fd\bar{3}m$	41.7	216
	$Ca_2Y_3H_{30}$	$R\bar{3}m$	5.1	249	YH_9 type*	Y_3ThH_{36}	$R3m$	32.6	224
	Ca_3LaH_{24}	$Fm\bar{3}m$	24.9	249		$YThH_{18}$	$P\bar{6}m2$	0	209
	Ca_2LuH_{18}	$I4/mmm$	1.0	249		$LaTh_3H_{36}$	$P\bar{4}3m$	45.1	204
	Y_3HfH_{24}	$Fm\bar{3}m$	42.6	248	$Fd\bar{3}m$-AB_2H_{17}*	Li_2NaH_{17}[14]	$Fd\bar{3}m$	26.7	372
	$CaYH_{12}$[15]	$Fd\bar{3}m$	3.8	248		$SrSc_2H_{17}$	$Fd\bar{3}m$	48.8	319
	YLu_3H_{24}[13]	$Fm\bar{3}m$	20.1	247	$A_2B_2H_{23}$*	$Ca_2Sr_2H_{23}$	$P4_2/mmc$	0	215
	NaY_4H_{30}	$R\bar{3}m$	6.0	245	$A_1B_1H_8$*	$Sr_1B_1H_8$	$Fm\bar{3}m$	32.3	212
	$NaYH_{12}$	$P\bar{3}m1$	17.0	245	AB_3H_{28}*[16]	$Sr_1Sc_3H_{28}$	$P6/mmm$	45.5	258
	$YLuH_{12}$[13]	$Fd\bar{3}m$	12.4	244	$I4/mcm$-AB_2H_{17}*[12]	$Li_1La_2H_{17}$	$I4/mcm$	0	209
	$Y_2Lu_3H_{30}$	$R\bar{3}m$	15.5	243	$F\bar{4}3m$-$A_2B_4H_{33}$*[14]	$La_2Mg_4H_{33}$	$F\bar{4}3m$	22.6	303
	YLu_2H_{18}[13]	$P\bar{3}m1$	13.3	242		$Sr_2Sc_4H_{33}$	$F\bar{4}3m$	32.0	298
	$CaLuH_{12}$	$Fd\bar{3}m$	10.3	242		$Li_4La_2H_{33}$	$F\bar{4}3m$	25.4	281
	$CaLuH_{12}$	$I4_1/amd$	6.7	242		$Li_4Y_2H_{33}$	$F\bar{4}3m$	16.2	252
	$YLuH_{12}$	$P\bar{3}m1$	9.2	242		$Ce_2Sc_4H_{33}$	$F\bar{4}3m$	37.8	251
	Y_3ScH_{24}	$Fm\bar{3}m$	33.3	241	R3m-$A_2B_4H_{33}$	$Li_4Ca_2H_{33}$	$R3m$	23.7	275
	Y_2LuH_{18}[13]	$P\bar{3}m1$	3.5	241	AB_3H_{39}	La_3YH_{39}	$R3m$	36.3	246
	$CaZrH_{12}$	$Fd\bar{3}m$	36.3	241		$SrTh_3H_{39}$	$R3m$	17.9	223
	$Ca_4Y_3H_{42}$	$R\bar{3}m$	1.8	241		$SrLa_3H_{39}$	$R3m$	32.2	212
	Y_3LuH_{24}	$P4/mmm$	4.3	240	$A_3B_4H_{41}$	$Na_3La_4H_{41}$	$I\bar{4}3m$	46.4	240
	Lu_3ZrH_{24}	$Fm\bar{3}m$	44.8	239		$Sr_4Mg_3H_{41}$	$I\bar{4}3m$	44.2	223
	$CaLu_2H_{18}$[13]	$P\bar{3}m1$	8.8	239	AB_4H_{28}	Lu_4SH_{28}	$P\bar{4}3m$	30.8	229
	$Y_3Lu_2H_{30}$	$R\bar{3}m$	10.4	239	$Pm\bar{3}m$-AB_4H_{32}	La_4SH_{32}	$Pm\bar{3}m$	13.9	251
	Ca_3YH_{24}	$P4/mmm$	4.5	239	$P\bar{4}3m$-AB_4H_{32}	Y_4BH_{32}	$P\bar{4}3m$	11.5	262
	Y_2LuH_{18}	$C2/m$	11.7	239	AB_4H_{28}	$Y_4H_{28}Br$	$P\bar{4}3m$	40.9	209
	YLu_4H_{30}	$R\bar{3}m$	21.3	239	$A_2B_4H_{49}$	$Y_2Sc_4H_{49}$	$P\bar{6}m2$	41.0	296
	$SrCa_3H_{24}$	$Fm\bar{3}m$	35.3	237	AB_2H_{28}	Y_2ThH_{28}	$P6_3mc$	18.4	255
	Ca_2LuH_{18}[13]	$P\bar{3}m1$	4.2	237	AB_3H_{32}	Ca_3YH_{32}	$R3m$	37.1	250
	Ca_3LuH_{24}	$P4/mmm$	3.2	237	$A_3B_3H_{58}$	$Sr_3Th_3H_{58}$	$R32$	20.7	244
	$CaLu_2H_{18}$	$Fmmm$	10.8	237	$A_3B_4H_{40}$	$Na_3Ca_4H_{40}$	$P6mm$	32.0	225
	Y_4ScH_{30}	$R\bar{3}m$	22.1	236	AB_3H_{34}	La_3LuH_{34}	$P\bar{3}m1$	39.6	218
	Ca_3YH_{24}[11]	$Fm\bar{3}m$	0	236	ABH_{13}	$LaYH_{13}$	$P3_221$	44.9	214
	Ca_3ScH_{24}	$R\bar{3}m$	14.1	236	AB_3H_{31}	YZr_3H_{31}	$P\bar{6}2m$	36.6	207
	$CaLu_3H_{24}$[13]	$Fm\bar{3}m$	16.9	234	$A_3B_3H_{49}$	$Na_3Lu_3H_{49}$	$R32$	20.8	207
	Ca_3LuH_{24}[13]	$Fm\bar{3}m$	0.4	233	$P6_3mc$-ABH_{19}	$LaThH_{19}$	$P6_3mc$	31.8	206
	Ca_3ScH_{24}	$Fm\bar{3}m$	16.8	232	$P6_3/mmc$-ABH_{19}	$LaThH_{19}$	$P6_3/mmc$	13.3	203
	Y_4ThH_{30}	$R\bar{3}m$	35.2	231	$P\bar{6}m2$-ABH_{19}	$YLuH_{19}$	$P\bar{6}m2$	49.2	276
	Ca_3MgH_{24}	$Fm\bar{3}m$	25.6	231	$A_3B_3H_{55}$	$Y_3Th_3H_{55}$	$R32$	0	202
	$Ca_3Lu_2H_{30}$	$R\bar{3}m$	4.4	231	$A_4B_2H_{55}$	$Y_4Lu_2H_{55}$	$R3$	20.5	230
	$Na_2Y_3H_{30}$	$R\bar{3}m$	14.3	229	AB_2H_{14}	Y_2BH_{14}	$R3m$	19.6	202
	Lu_3ScH_{24}	$Fm\bar{3}m$	49.4	228	$A_4B_3H_{60}$	$Th_4Mg_3H_{60}$	$Im\bar{3}$	37.2	201
	$Y_4Lu_3H_{42}$	$R\bar{3}m$	6.6	224	$A_4B_2H_{31}$	$Li_4Sr_2H_{31}$	$R3m$	20.0	262
	Y_3ThH_{24}	$Fm\bar{3}m$	40.5	222	$A_1B_2H_{22}$	$LaLu_2H_{22}$	$C2/m$	15.9	240
	$NaLu_2H_{18}$	$P\bar{3}m1$	15.0	219	$A_2B_4H_{35}$	$Y_4Lu_2H_{35}$	$P\bar{4}m2$	18.9	225
	Ca_4YH_{30}	$R\bar{3}m$	0.7	219	$A_3B_3H_{59}$	$Y_3La_3H_{59}$	$R32$	12.2	200

Section SIV. Detailed result of twelve compounds exhibiting high T_cs

The detailed EPC calculation results of the twelve compounds: $Fd\bar{3}m$-$SrSc_2H_{17}$ ($T_c \approx 319$ K), $Fd\bar{3}m$-$SrLu_2H_{16}$ ($T_c \approx 319$ K), $F\bar{4}3m$-$La_2Mg_4H_{33}$ ($T_c \approx 303$ K), $Pm\bar{3}m$-Y_3ThH_{40} ($T_c \approx 302$ K), $F\bar{4}3m$-$Sr_2Sc_4H_{33}$ ($T_c \approx 298$ K), $P\bar{6}m2$-$Y_2Sc_4H_{49}$ ($T_c \approx 296$ K), $Fd\bar{3}m$-$SrYH_{12}$ ($T_c \approx 291$ K), $P6/mmm$-Y_2ThH_{24} ($T_c \approx 291$ K), $Fd\bar{3}m$-Li_2NaH_{17} ($T_c \approx 372$ K), $R\bar{3}m$-$Sr_3Y_4H_{42}$ ($T_c \approx 308$ K), $Fm\bar{3}m$-KLu_3H_{24} ($T_c \approx 301$ K), and $R\bar{3}m$-Y_3ThH_{40} ($T_c \approx 300$ K) are illustrated in Figure **S2**. These are compounds predicted to exhibit T_c approaching or exceeding room temperature. For each compound, the phonon dispersion spectrum, projected phonon density of states, Eliashberg epectral function $\alpha^2F(\omega)$ and EPC constant λ, and the superconducting gap with μ^* ranging from 0.10-0.16 are shown in panels from the left to the right.

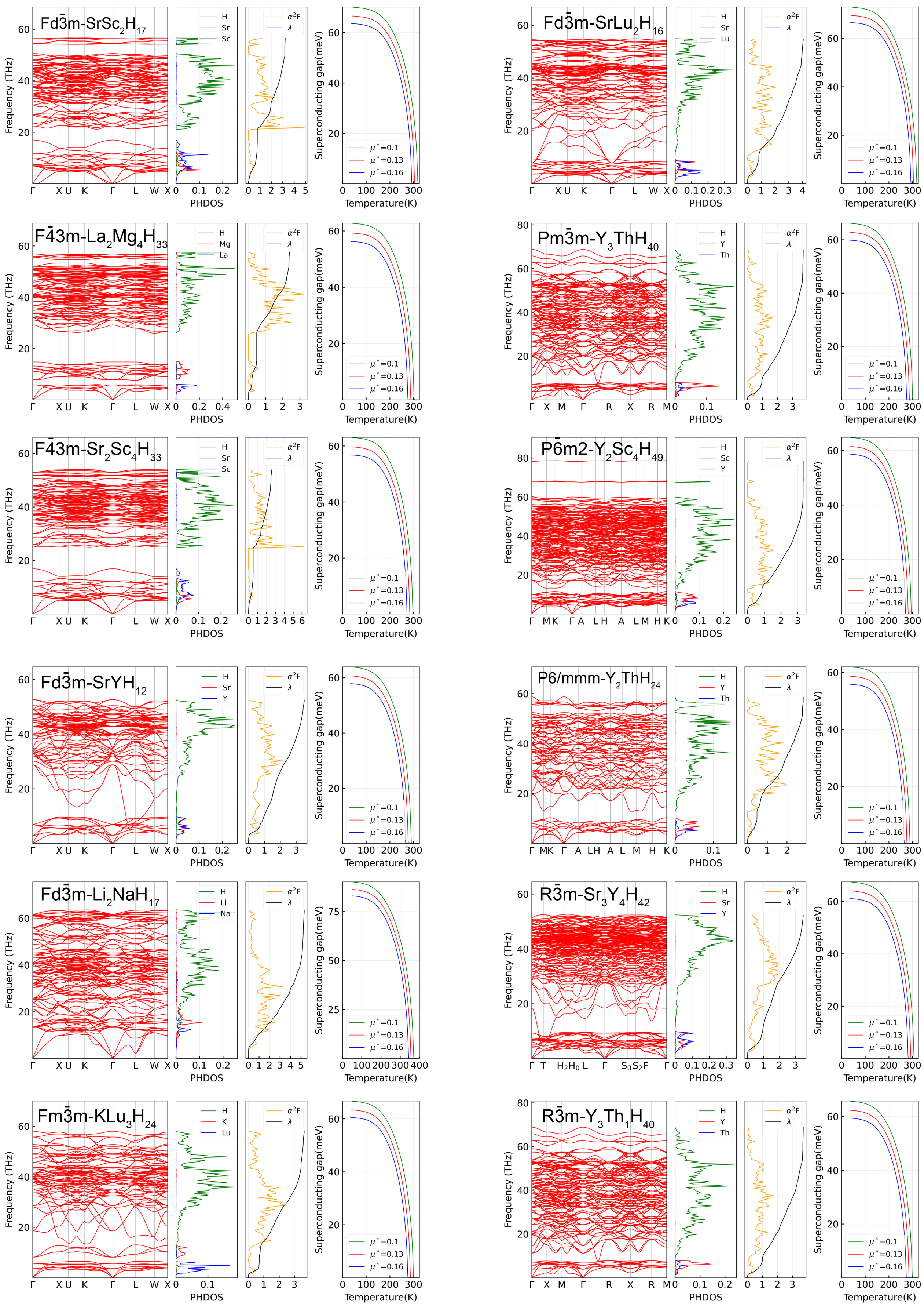

Figure S2. The EPC calculation results of compounds with T_c approaching or exceeding room temperature.

[1] Z. Wang, X. Wang, X. Luo, P. Gao, Y. Sun, J. Lv, H. Wang, Y. Wang, and Y. Ma, Concurrent learning scheme for crystal structure prediction, Physical Review B **109**, 094117 (2024).

[2] Y. Zhang, H. Wang, W. Chen, J. Zeng, L. Zhang, H. Wang, and E. Weinan, DP-GEN: A concurrent learning platform for the generation of reliable deep learning based potential energy models, Computer Physics Communications **253**, 107206 (2020).

[3] A. E. Mattsson, *A lithium projector augmented wave potential suitable for use in VASP at high compression and temperature.*, Tech. Rep. (Sandia National Laboratories (SNL), Albuquerque, NM, and Livermore, CA ..., 2012).

[4] D. Zhang, H. Bi, F.-Z. Dai, W. Jiang, X. Liu, L. Zhang, and H. Wang, Pretraining of attention-based deep learning potential model for molecular simulation, npj Computational Materials **10**, 94 (2024).

[5] H. Wang, L. Zhang, J. Han, and E. Weinan, Deepmd-kit: A deep learning package for many-body potential energy representation and molecular dynamics, Computer Physics Communications **228**, 178 (2018).

[6] D. Kingma and J. Ba, Adam: a method for stochastic optimization, in *Proceedings of the International Conference on Learning Representations (ICLR)* (2015).

[7] A. Hjorth Larsen, J. Jørgen Mortensen, J. Blomqvist, I. E. Castelli, R. Christensen, M. Dułak, J. Friis, M. N. Groves, B. Hammer, C. Hargus, E. D. Hermes, P. C. Jennings, P. Bjerre Jensen, J. Kermode, J. R. Kitchin, E. Leonhard Kolsbjerg, J. Kubal, K. Kaasbjerg, S. Lysgaard, J. Bergmann Maronsson, T. Maxson, T. Olsen, L. Pastewka, A. Peterson, C. Rostgaard, J. Schiøtz, O. Schütt, M. Strange, K. S. Thygesen, T. Vegge, L. Vilhelmsen, M. Walter, Z. Zeng, and K. W. Jacobsen, The atomic simulation environment—a python library for working with atoms, Journal of Physics: Condensed Matter **29**, 273002 (2017).

[8] D. V. Semenok, W. Chen, X. Huang, D. Zhou, I. A. Kruglov, A. B. Mazitov, M. Galasso, C. Tantardini, X. Gonze, and A. G. Kvashnin, Sr-doped superionic hydrogen glass: Synthesis and properties of srh22, Advanced Materials **34**, 2200924 (2022).

[9] Q. Jiang, D. Duan, H. Song, Z. Zhang, Z. Huo, S. Jiang, T. Cui, and Y. Yao, Prediction of room-temperature superconductivity in quasi-atomic h2-type hydrides at high pressure, Advanced Science **11**, 2405561 (2024).

[10] Y. Sun, Y. Wang, X. Zhong, Y. Xie, and H. Liu, High-temperature superconducting ternary Li$-R-$H superhydrides at high pressures ($R = \mathrm{Sc, Y, La}$), Physical Review B **106**, 024519 (2022).

[11] W. Zhao, D. Duan, M. Du, X. Yao, Z. Huo, Q. Jiang, and T. Cui, Pressure-induced high$-T_c$ superconductivity in the ternary clathrate system Y-Ca-H, Physical Review B **106**, 014521 (2022).

[12] B. Jiang, X. Luo, Y. Sun, X. Zhong, J. Lv, Y. Xie, Y. Ma, and H. Liu, Data-driven search for high-temperature superconductors in ternary hydrides under pressure, Physical Review B **111**, 054505 (2025).

[13] M. Du, H. Song, Z. Zhang, D. Duan, and T. Cui, Room-Temperature Superconductivity in Yb/Lu Substituted Clathrate Hexahydrides under Moderate Pressure, Research **2022**, 9784309 (2022).

[14] D. An, L. J. Conway, D. Duan, Z. Zhang, Q. Jiang, H. Song, Z. Huo, C. J. Pickard, and T. Cui, Prediction of thermodynamically stable room-temperature superconductors in li-na hydrides under high pressure, Advanced Functional Materials , 2418692 (2024).

[15] X. Liang, A. Bergara, L. Wang, B. Wen, Z. Zhao, X.-F. Zhou, J. He, G. Gao, and Y. Tian, Potential high-T_c superconductivity in $CaYH_{12}$ under pressure, Physical Review B **99**, 100505 (2019).

[16] G. M. Shutov, D. V. Semenok, I. A. Kruglov, and A. R. Oganov, Ternary superconducting hydrides in the La–Mg–H system, Materials Today Physics **40**, 101300 (2024).
